# Fertility-enhancing hysteroscopic surgery; multi-center retrospective cohort study of reproductive outcome

**DOI:** 10.1186/s12905-023-02562-2

**Published:** 2023-08-29

**Authors:** Naser Al-Husban, Omar Odeh, Muataz AlRamahi, Sara Qadri, Hedaieh Al-Husban

**Affiliations:** 1 School of Medicine, The University of Jordan, P O Box 2194, Amman, 11941 Jordan; 2 Jordan University hospital, Amman, Jordan; 3 Al-Noor Fertility Centre, Amman, Jordan; 4 Arab Medical Centre, Amman, Jordan

**Keywords:** Infertility, Hysteroscopy, Reproduction, Septoplasty myomectomy

## Abstract

**Introduction:**

Hysteroscopic surgery and assisted reproduction technology are feasible ways to improve the reproductive outcome. Our aim was to study hysteroscopic septoplasty and myomectomy’s effect on infertility and reproductive performance.

**Methods:**

Retrospective cohort of patients who had unexplained infertility and/or recurrent miscarriages and had myomectomy or septoplasty in the period September 2016-october 2021 with a total of 18 months’ follow up. The main outcome measures were spontaneous pregnancy, term pregnancy and miscarriage. For analysis, we used Statistical Package for Social Sciences (SPSS) version 20.

**Results:**

One hundred and sixty-five patients were included. The mean age of patients was 39 years. 40 patients had septum resection and 125 patients had hysteroscopic myomectomy. A spontaneous pregnancy rate after surgery was achieved in 46 patients (27.9%). Out of the 64 patients who had failed IVF preoperatively, 32 patients (50%) had a successful IVF post-hysteroscopic surgery and there were more successful cases in the patients who had fibroid resection but this difference did not reach a statistical significance (P value 0.055). In the 79 pregnancies after surgery, preterm birth and miscarriage were seen in 10 patients (12.7%), similarly, respectively after septal or fibroid resection. Miscarriages were less post-operatively. Hysteroscopic myomectomy, compared with hysteroscopic metroplasty, was significantly associated with higher spontaneous pregnancy rate (63.0% Vs 37.0%, P value 0.018), more term pregnancies (87.5% vs. 12.5%, P value 0.001) and less miscarriage rate (40%vs 60%, P value 0.003). Pregnancy post-operatively in patients with primary infertility was more statistically significantly associated with hysteroscopic myomectomy than with hysteroscopic septoplasty (95.8% vs. 4.2%, p value 0.030). In patients who got pregnant postoperatively there was no statistically significant difference in the mode of delivery.

**Conclusion:**

In carefully selected patients with unexplained infertility and recurrent miscarriage, hysteroscopic myomectomy, compared with hysteroscopic metroplasty, was significantly associated with higher spontaneous pregnancy, more term pregnancies and less miscarriage rates. More than metroplasty, hysteroscopic myomectomy led to higher spontaneous pregnancies in patients with primary infertility.

**Trial registration:**

NCT05560295.

## Introduction

Septate uterus is a common benign uterine congenital anomaly with an estimated prevalence of ~ 2% in the general population, ~ 3% in infertile women and > 5% in patients with recurrent pregnancy losses [1]. A critical analysis of studies suggests that the prevalence of congenital uterine anomalies is approximately 6.7% [95% confidence interval (CI), 6.0-7.4] in the general population, approximately 7.3% (95% CI, 6.7–7.9) in the infertile population and approximately 16.7% (95% CI, 14.8–18.6) in the RM population [2]. The prevalence of septate uterus in a non-selected population varies significantly depending the diagnostic criteria used [3].

Hysteroscopic septum resection in women with septate uterus significantly improved the live birth rates and future fertility was not impaired [4]. A hospital-based study of infertile women who had undergone transvaginal hysteroscopy and used different treatment protocols after hysteroscopic correction of uterine septum found that it was a safe and an efficacious procedure. Treatment following hysteroscopic septum resection, either the consistent or the alternate protocol was both beneficial to improve pregnancy rate [5]. Moreover, hysteroscopic septum resection was found to be a safe and effective method for patients with history of infertility or recurrent miscarriage [6]. Clinical evidence from the studies analyzed suggested an improvement in reproductive outcomes after hysteroscopic resection of the septum, particularly in infertile women and women who have experienced recurrent miscarriages [7].

Hysteroscopic myomectomy was also found to be a safe procedure to enhance fertility especially in cases with unexplained infertility [8].

When immediate fertility is a priority and in the presence of more than 1 asymptomatic submucous myomas ≥ 5 mm, hysteroscopic myomectomy is recommended [9]. When immediate fertility is not desired and in the presence of more than 1 asymptomatic submucous myoma smaller than 15 mm, hysteroscopic myomectomy is recommended, but expectant management is acceptable. If expectant management is favored, clinical surveillance of symptoms and serial transvaginal pelvic ultrasounds to monitor growth of the myomas are recommended [9].

Nowadays, the hysteroscopic approach to treat these cases are the only feasible way to improve their reproductive outcome [28].

Our study’s objectives were to investigate the fertility enhancing effects of two types of hysteroscopic surgery; namely septoplasty and myomectomy in patients with an otherwise unexplained infertility and the pregnancy outcomes in these patients. We also aimed to find out which of these types of surgery had greater benefit than the other.

## Materials and methods

### Study design

Historical cohort study of the reproductive outcome in patients who underwent hysteroscopic surgery for otherwise unexplained infertility, those who had recurrent miscarriages and those who had preterm birth. Patients had a minimum of 6 months follow up after surgery for a spontaneous pregnancy to occur. The total period of follow up was 18 months and those who conceived were followed up until delivery.

#### Settings

Jordan university hospital and Al-Noor fertility center in Amman, Jordan in the period September 2016-october 2021.

### Population

Patients who had uterine septum or submucosal fibroids were included. Only patients 18–45 years old with at least one year infertility were included. They had normal seminal fluid analysis, normal female hormonal profile, patent fallopian tubes bilaterally (as confirmed by hysterosalpingography HSG), normal thyroid function, no previous uterine surgery, normal anti-mullerian hormone (AMH) levels and no medical diseases. Included cases with recurrent miscarriages are those with 2 or more first or second trimester miscarriages of an apparently normal singleton fetus with no hormonal, endocrine or maternal or paternal karyotype abnormalities and with no previous history of term pregnancy(s).

Uterine septum diagnosis was confirmed by HSG, diagnostic hysteroscopy and diagnostic laparoscopy to confirm the diagnosis and exclude other uterine anomalies and endometriosis. Patients with endometriosis were excluded from the study whether they had uterine septum or fibroid.

Submucosal uterine fibroids were diagnosed using trans-vaginal ultrasound scans (TV-US) and confirmed by diagnostic hysteroscopy. Only patients with lesions larger than 0.5 cm and less than 6 cm were included. Only patients with uterine submucosal fibroids types 0 and 1 were included. These were pedunculated intracavitary (type 0) and less than 50% intramural (type 1) [10–12].

Regarding uterine septum, all patients had TV-US, HSG, diagnostic hysteroscopy and diagnostic laparoscopy to confirm the diagnosis and to exclude bicornuate uterus and other congenital anomalies. All procedures were done by only two consultant gynecologists, one at each center, and all procedures were timely done post-menstrual. Most procedures were performed using monopolar hysteroscopic instruments. For the monopolar instruments, we used 3.0-L bags of 1.5% glycine as a distending medium. The cervix was gradually dilated up to 10 mm using Hegar’s dilators. We did not use preoperative endometrial thinning agents or cervical preparation agents. All patients had a standardized intrauterine pressure of 80 mmHg during surgery using the same automated glycine infusion pump. We used passive handle mechanism, continuous flow 30 degrees hysteroscopic loop for the fibroids and knife electrode for the septal resection.

We did not use adhesion barriers post-operatively as they were not available in our country. All patients were given prophylactic antibiotics pre-operatively. Most of our procedures were performed under general with a small proportion done under spinal anesthesia. The choice of the type of anesthesia was left to the anesthetist. All patients were prescribed postoperative prophylactic antibiotics. All patients were offered HSG or second look hysteroscopy after the first post-operative menstrual period to confirm complete resection and absence of possible intra-uterine adhesions. In addition, those patients with large fibroids and incomplete resection had their resection completed in the second session. The patients were followed up for spontaneous pregnancy, success of IVF and pregnancy outcome (miscarriage, preterm and term). The main outcome measures were spontaneous pregnancy, term pregnancy and miscarriage. The primary outcomes were spontaneous pregnancy and success of assisted reproduction. The secondary outcomes were pregnancy outcome (miscarriage, preterm and term delivery).

### Exclusion criteria

All patients who were operated upon outside the context of infertility (heavy bleeding…etc.), patients with combined (maternal and paternal) infertility factors, patients with history of any number of term pregnancy, previous uterine surgery, patients with medical diseases, those with abnormal female hormonal profile, those with other potential factors for infertility including endometriosis (excluded by both negative clinical symptoms and normal diagnostic laparoscopy) and adenomyosis, and patients with incomplete or missing data and could not be contacted. Those with unexplained primary or secondary infertility were excluded. These exclusion criteria allowed to study the effect of such hysteroscopic surgery only. For homogeneity of the procedure, patients who had myomectomy or septal resection done using bipolar hysteroscopy were also excluded.

### Data collection

Data was obtained from the electronic and paper-based clinical files for patients who were operated upon at Jordan University hospital. Data of those cases who had their operation at Al-Noor fertility center were obtained from paper-based files. Patients who missed follow up after the operation were contacted by phone or invited them for a clinic review to know of their menstrual pattern, fertility status and pregnancy outcome. Data that were collected included hospital identification number (ID), age, parity, miscarriages, previous uterine surgery (myomectomy), past medical history, past surgical history, history of uterine curettage, clinical symptoms leading to the current diagnosis, indication of the hysteroscopy, failure of intra-uterine insemination (IUI), failure of in vitro fertilization (IVF) implantation failure, recurrent first and second trimester miscarriages and preterm/term deliveries.

### Statistical methodology

We used Statistical Package for Social Sciences (SPSS) version 20. Analysis was done in two phases. In the first one we assessed the difference in the effect between the removal of septoplasty and myomectomy on various obstetric outcomes after surgery, and these variables included; occurrence of a spontaneous pregnancy, resumption of menses, if no conception was achieved, if a trial of IVF was done, the result of IVF if a trial was done, if the patient got pregnant regardless of her history, the number of term births, preterm births, and miscarriages in the patients who got pregnant, and the delivery details whether it was a normal vaginal or operative delivery). The second phase of analysis was done with the purpose of assessing the difference in the effect of both surgeries on fertility, where we split our study population into 3 categories: Patient with primary infertility, patients with secondary infertility, and patients with infertility and secondary symptoms. To carry out statistical analysis, a Pearson Chi Squared test (for categorical variables) was done, and a p-value of less than 0.05 was deemed significant and using the percentages, counts, and expected counts, we interpreted the p-values to find out exactly where the significance lies. A T-test was used to compare continuous variables. The study was powered for the primary outcome. Sample size was calculated to be 100 subjects with a 90% power and 0.05 alpha.

### Ethical approval and consent to participate

The study obtained an institutional review board (IRB) approval from the committee at Jordan University hospital, number 171/2020 dated 23/06/2020. informed consents from the patients were waived by this committee due to retrospective nature.

Patients’ data was treated with the utmost of confidentiality. Any and all data that contributed to the recognition of patients’ identities, such as names, phone numbers or addresses were either turned into codes or not be used at all.

It was also registered in clinicaltrials.gov; ClinicalTrials.gov Identifier: NCT05560295.

### Consent for publication

Not applicable.

## Results

### Descriptive data

One hundred and sixty-five patients were finally included in the study. This was due to the rigorous and wide exclusion criteria to obtain a relatively homogenous population. The mean age of the patients was 39 years. The mean AMH level was 1.43 ng/ml with a range of 1.0-2.5 ng/ml. Our laboratory normal range for AMH was 1.0–4.0 ng/ml. Most of the patients before surgery were nulliparous (101 patients, 61.2%). Septum resection was performed in 40 patients (24.2%) and hysteroscopic myomectomy was done in 125 patients (75.8%) (Table 1).

Among patients with uterine septum, there were 8 patients with partial (class U2a) and 32 patients with complete septum (class U2b) according to the ESHRE/ESGE classification [11].

Four patients with large fibroids required another hysteroscopic myomectomy as complete resection was not achieved in the first session.

### Outcome data and main results

Of all patients who had surgery, 162 patients (98.2%) resumed normal menstrual cycles post-operatively. The overall pregnancy rate after the two types of surgery was 79 pregnancies (47.9%). A spontaneous pregnancy rate after surgery was achieved in 46 patients (27.9%). Out of the 64 patients who had failed IVF preoperatively, 32 patients (50%) had a successful IVF post-hysteroscopic surgery. Regardless of the type of surgery, most of the pregnancies achieved were term pregnancies (70.9%). In the 79 pregnancies after surgery, preterm birth and miscarriage were seen in 10 patients (12.7%), similarly, respectively after septal or fibroid resection. Miscarriages were 19 pre-operatively and only 10 post-operatively (Table 1).

**Table 1:** Description of the population included in the study

		Mean ± SD	N	%
Age (N = 165)		39 ± 3.5		
Parity (prior to fertility enhancing surgery)	PARA 0		101	61.2%
	PARA 1–3		45	27.3%
	PARA > 3		19	11.5%
Indication for surgery	Septum		40	24.2%
	fibroid		125	75.8%
Primary infertility	No		106	64.2%
	Yes		59	35.8%
Secondary infertility	No		109	66.1%
	Yes		56	33.9%
Recurrent miscarriages	No		146	88.5%
	Yes		19	11.5%
Infertility with other symptoms	No		136	82.4%
	Yes		29	17.6%
Postop conception	No		133	80.6%
	Yes		32	19.4%
Resumption of normal menses postop	No		2	1.2%
	Yes		163	98.8%
Postop spontaneous pregnancy	No		119	72.1%
	Yes		46	27.9%
Patients tried IVF postop	No		101	61.2%
	Yes		64	38.8%
Result for trial of IVF (n = 64)	Failed		32	50.0%
	Successful		32	50.0%
The patients get pregnant after the surgery (regardless of history) (n = 165)	No		86	52.1%
	Yes		79	47.9%
Term births in patients who got pregnant after surgery (N = 79)	No		23	29.1%
	Yes		56	70.9%
Preterm births in patients who got pregnant after surgery (N = 79)	No		69	87.3%
	Yes		10	12.7%
Miscarriage in patients who got pregnant after surgery (N = 79)	No		69	87.3%
	Yes		10	12.7%
Operative Delivery in patients who got pregnant after surgery (N = 79)	No		39	49.4%
	Yes		40	50.6%
Normal Vaginal Delivery in patients who got pregnant after surgery (N = 79)	No		50	63.3%
	Yes		29	36.7%

There was a total of 165 patients, SD; standard deviation, N; number, %; per cent, IVF; in vitro fertilization

Hysteroscopic myomectomy, compared with hysteroscopic metroplasty, was significantly associated with higher spontaneous pregnancy rate (63.0% Vs 37.0%, P value 0.018), more term pregnancies (87.5% vs. 12.5%, P value 0.001) and less miscarriage rate (40%vs 60%, P value 0.003). In the 64 patients who tried IVF post-operatively, there were more successful cases in the patients who had fibroid resection but this difference did not reach a statistical significance (p value 0.055). In patients who got pregnant postoperatively (79 patients), there was no statistically significant difference in the normal and operative delivery (p value 0.371 and 0.257, respectively) (Table 2).

**Table 2:** Comparing the effect of Septum resection vs. fibroid resection on various postop

VARIABLE		Indication for surgery (Septum or fibroid)				p value
		Septum		Fibroid		
		N	%	N	%	
Spontaneous Pregnancy postop (N = 165)	No	23	19.3%	96	80.7%	0.018*
	Yes	17	37.0%	29	63.0%	
Resumption of menses postop (N = 165)	No	1	50.0%	1	50.0%	0.369
	Yes	40	24.2%	123	75.9%	
No conception (N = 165)	No	31	23.3%	102	76.7%	0.568
	Yes	9	28.1%	23	71.9%	
Tried IVF postop (N = 165)	No	28	27.7%	73	72.3%	0.190
	Yes	12	18.8%	52	81.3%	
IVF result if trial was done (N = 64)	Failed	9	28.1%	23	71.9%	0.055
	Successful	3	9.4%	29	90.6%	
Did the patient get pregnant after the surgery regardless of history (N = 165)	No	22	25.6%	64	74.4%	0.675
	Yes	18	22.8%	61	77.2%	
Term births in patients who got pregnant after surgery (N = 79)	No	11	47.8%	12	52.2%	> 0.001*
	Yes	7	12.5%	49	87.5%	
Preterm births in patients who got pregnant after surgery (N = 79)	No	13	18.8%	56	81.2%	0.028*
	Yes	5	50.0%	5	50.0%	
Miscarriage in patients who got pregnant after surgery (N = 79)	No	12	17.4%	57	82.6%	0.003*
	Yes	6	60.0%	4	40.0%	
Operative Delivery in patients who got pregnant after surgery (N = 79)	No	11	28.2%	28	71.8%	0.257
	Yes	7	17.5%	33	82.5%	
Normal Vaginal Delivery in patients who got pregnant after surgery (N = 79)	No	13	26.0%	37	74.0%	0.371
	Yes	5	17.2%	24	82.8%	

* The results are significant as p value is < 0.05, There was a total of 165 patients, N; number, %; per cent, IVF; in vitro fertilization

Achieving a pregnancy post-operatively in patients with primary infertility was more statistically significantly associated with hysteroscopic myomectomy than with hysteroscopic metroplasty (95.8% vs. 4.2%, p value 0.030). this difference was not seen in cases of secondary infertility (p value 0.478) (Table 3).

**Table 3:** Effect of surgery on infertility

Variable		Indication for surgery (Septum or fibroid)				p value
		Septum		Fibroid		
		N	%	N	%	
Was pregnancy achieved in patients with primary infertility after surgery? (N = 59)	No	9	25.7%	26	74.3%	0.030*
	Yes	1	4.2%	23	95.8%	
Was pregnancy achieved in patients with secondary infertility after surgery? (N = 56)	No	6	17.0%	17	83.0%	0.478
	Yes	6	27.0%	27	73.0%	
Was a pregnancy achieved in patients with infertility and other symptoms? (N = 29)	No	4	16.7%	20	83.3%	0.858
	Yes	1	20.0%	4	80.0%	

* The results were significant as p value was < 0.05

There were only 2 cases complicated by excessive glycine absorption in patients who had resection of 5–6 cm type 1 fibroids. They were managed with diuretics and normal saline infusion. Both went home on the day following the surgery. There were no other complications. There were no intrauterine adhesions found post-operatively whether in the second look hysteroscopy or reported in the HSG.

## Discussion

### Findings and interpretation

The enhanced fertility potential that was suggested by our study carried a unique significance as it involved 2 clinical situations namely uterine septum and submucosal fibroids. Moreover, the ESHRE/ESGE classification of the uterine anomalies was followed by the 2 consultant gynecologists who operated on the patients [13]. The effect of hysteroscopic resection of these lesions was investigated in relation to infertility and pregnancy outcome. Although the mean age of our patients was 39, the study showed a post-operative IVF success rate in previously failed attempts to be 50%. A small partial uterine septum was found to be an important and preventable risk factor for spontaneous abortion in pregnancies after IVF and ICSI [14]. In addition, nearly half of the patients who had resection of U1a and U2 uterine anomalies achieved a spontaneous pregnancy and this carried promising results in unexplained infertility and repeated IVF/ICSI failures [15]. In our study a spontaneous pregnancy rate was seen in 27.9% over 6 months-follow up. Hysteroscopic metroplasty might contribute to improved IVF outcomes even in patients with T-shaped uterus [16].

Most of our patients had normal menstrual cycles post-operatively. There was also an obvious improvement in IVF implantation rate and reduction in miscarriage and preterm birth rates. Hysteroscopic septum resection was accompanied by safe improvement in reproductive performance in infertility and miscarriage patients [17].

Miscarriage rate in our study was seen only in 10 cases out of 79. Patients obtained a significant improvement of reproductive outcomes with a fivefold reduction in miscarriage rate after hysteroscopic septoplasty [18].

Our study included cases with well-defined septum as judged by the consultants which may have contributed to the obvious enhancing surgical effect. Women with a septum size larger than one-half of their uterine length were found to have a higher chance of successful pregnancy after hysteroscopic resection [19].

The follow up period in our study was a minimum of 6 months with a very good spontaneous pregnancy rate and term pregnancy rate. Although a small-sized study, women with septate uterus and unexplained infertility could have improved clinical pregnancy rate and live birth rate after resection and a spontaneous pregnancy was more likely to occur during the first 15 months following the procedure [20]. The reduction in miscarriage rate and preterm birth rate in our study was consistent with those found in a systematic review and meta-analysis [21]. Our findings regarding hysteroscopic septoplasty were also consistent with those found in a Systematic Review and Meta-Analysis of Observational Research [22]; a detrimental effect of the uterine septum was found on pregnancy rate, low birth rate, spontaneous abortion and preterm labor. Its treatment reduced the rate of spontaneous abortion [22].

In our study, there was a reduction in the IVF implantation failure rate. Half of the patients who repeated their IVF following hysteroscopic septoplasty or myomectomy, after at least 2 previous failed attempts, had a successful implantation. The reproductive outcomes of IVF/ICSI after septum resection were better than that in the untreated group, suggesting that septum resection with infertility improved the reproductive performance [23]. Our post-septal resection IVF findings were also supported by a recent literature review which concluded that in cases of recurrent implantation failure or recurrent pregnancy loss after IVF, septoplasty could be proposed and they also recommended hysteroscopic septum incision in primary infertility and for patients undergoing assisted reproductive technologies [24].

Our study included 125 patients who had hysteroscopic myomectomy. It was shown that myomectomy had significant enhancing effects on reproductive performance of these patients. Hysteroscopic resection of submucous fibroids was shown to improve the cumulative pregnancy rate and the live birth rate in women with a history of reproductive failure [25]. Hysteroscopic myomectomy for submucosal fibroids but not type 2 had proven capable of increasing fertility rates [26]. This explained our results as our patients had only type 0 or 1 submucosal fibroids. Concerning other types of fibroids, the existing evidence until 2020 needed to be viewed with caution due to the small number of events, minimal number of studies and very low-quality evidence [27]. In these patients the probability of conception or live birth did not differ appreciably by the myomectomy route among women observed for 36 months postoperatively [28]. In our study, the pregnancy outcome was shown to be improved after hysteroscopic myomectomy, namely miscarriage and preterm birth as myomas that distort the uterine cavity and larger intramural myomas were associated with not only infertility but also with an increased risk of spontaneous abortion, preterm delivery and others [29]. Moreover, a strong association was found between clinically significant uterine fibroids and preterm birth but not postpartum hemorrhage (PPH), placental abruption or IUGR [30]. The preterm birth rate in our study was significantly reduced after hysteroscopic myomectomy. Despite fibroids were found to be associated with an increased risk of preterm birth and with a stronger risk at earlier gestational ages [31], a prospective cohort study encouraged a reconsideration of the clinical impression that presence of fibroids was a major risk factor for preterm birth [32]. However, a systematic review and meta-analysis found higher risk for preterm birth and other adverse obstetric outcomes in women with uterine fibroids and advised for closer monitoring [33].

### Similarities in relation to other studies

We found that IVF implantation rate was improved following hysteroscopic myomectomy. That effect was greater with myomectomy than septoplasty. Available evidence from IVF studies supported a detrimental effect of submucosal and intramural fibroids on embryo implantation [34]. In women with otherwise unexplained infertility, submucosal fibroids should be removed in order to improve conception and pregnancy rates [35].

### Differences in our study

Our results showed that hysteroscopic myomectomy, compared with hysteroscopic septoplasty, had higher spontaneous pregnancy rate, more term pregnancies and less miscarriage rate. Also, our study did not show a statistically significant difference in mode of delivery between myomectomy and septoplasty. Although Hysteroscopic resection could effectively reduce the risk of abortion and malpresentation and increase term delivery rate, the rates of preterm birth, cesarean section, and postpartum hemorrhage after resection did not return to normal levels [36]. The benefits of septal resection on subfertility and pregnancy outcome were shown in some studies to be limited ( 37, 38). However, one study found that previous hysteroscopic resection of septum could be not considered a risk factor for preterm delivery in singleton pregnancies, irrespective of the modality of conception [39]. Moreover, uterine rupture in pregnancy after a previous septal resection was very rare in a 20-year retrospective analysis [40]. Lastly, septal dissection was not associated with higher rate of placental abnormalities in the first singleton pregnancy compared with other hysteroscopic procedures [41].

### Strengths and limitations of our study

The strengths of this study were in the highly selected population and the consistency in diagnosis and management of the patients. However, it was limited by the relatively small size and short duration of post-operative follow up.

Unanswered questions and future research; the effect of hysteroscopic resection of short uterine septum and intramural fibroids that do not disrupt the endometrial cavity needs to be investigated in well-designed randomized controlled studies.

Within the context of reproduction, our findings suggest that careful evaluation and classification of uterine pathologies are needed. Moreover, establishing regional or national guidelines and protocols with emphasis on training are of paramount significance.

Our study excluded those who had endometrial cancer. These patients need risk-stratification approach in line with fertility preservation. Molecular and genomic profiling might be useful to tailor the most appropriate adjuvant strategies in apparent early-stage EC [42]. Detecting and validating use of molecular classification in precancerous lesions and analyzing different molecular markers may change therapeutic strategy, increasing the follow up of fertility-sparing patients reserving demolition surgery only for patients at high risk of cancer progression [43].

## Conclusion

In carefully selected patients with unexplained infertility and recurrent miscarriage, hysteroscopic myomectomy, compared with hysteroscopic metroplasty, was significantly associated with higher spontaneous pregnancy, more term pregnancies and less miscarriage rates. More than metroplasty, hysteroscopic myomectomy led to higher spontaneous pregnancies in patients with primary infertility.

## Data Availability

The datasets used and/or analyzed during the current study available from the corresponding author on reasonable request.

## Abbreviations

D&C: Dilation and curettage
EP: Endometrial polyps
HSG: Hysterosalpingography
TV-US: Trans-vaginal ultrasound scans
ESGE: European society for gynecological endoscopy
ID: Identification number
IUI: Intra-uterine insemination
IVF: In vitro fertilization
ICSI: Intracytoplasmic Sperm Injection
IRB: Institutional review board
ESHRE: European Society of Human Reproduction and Embryology
